# Decitabine-induced DNA methylation-mediated transcriptomic reprogramming in human breast cancer cell lines; the impact of DCK overexpression

**DOI:** 10.3389/fphar.2022.991751

**Published:** 2022-10-05

**Authors:** Verona Buocikova, Silvia Tyciakova, Eleftherios Pilalis, Chara Mastrokalou, Maria Urbanova, Miroslava Matuskova, Lucia Demkova, Veronika Medova, Eleonora Marta Longhin, Elise Rundén-Pran, Maria Dusinska, Ivan Rios-Mondragon, Mihaela Roxana Cimpan, Alena Gabelova, Andrea Soltysova, Bozena Smolkova, Aristotelis Chatziioannou

**Affiliations:** ^1^ Cancer Research Institute, Biomedical Research Center of the Slovak Academy of Sciences, Bratislava, Slovakia; ^2^ e-NIOS Applications P.C., Athens, Greece; ^3^ Institute of Clinical and Translational Research, Biomedical Research Center, Slovak Academy of Sciences, Bratislava, Slovakia; ^4^ Health Effects Laboratory, NILU-Norwegian Institute for Air Research, Kjeller, Norway; ^5^ Department of Clinical Dentistry, University of Bergen, Bergen, Norway; ^6^ Department of Molecular Biology, Faculty of Natural Sciences, Comenius University in Bratislava, Bratislava, Slovakia; ^7^ Center of Systems Biology, Biomedical Research Foundation of the Academy of Athens, Athens, Greece

**Keywords:** decitabine, DNA methylation, gene expression, deoxycytidine kinase, cytosine deaminase, whole-genome analysis, epigenetic therapy

## Abstract

Decitabine (DAC), a DNA methyltransferase (DNMT) inhibitor, is tested in combination with conventional anticancer drugs as a treatment option for various solid tumors. Although epigenome modulation provides a promising avenue in treating resistant cancer types, more studies are required to evaluate its safety and ability to normalize the aberrant transcriptional profiles. As deoxycytidine kinase (DCK)-mediated phosphorylation is a rate-limiting step in DAC metabolic activation, we hypothesized that its intracellular overexpression could potentiate DAC’s effect on cell methylome and thus increase its therapeutic efficacy. Therefore, two breast cancer cell lines, JIMT-1 and T-47D, differing in their molecular characteristics, were transfected with a DCK expression vector and exposed to low-dose DAC (approximately IC_20_). Although transfection resulted in a significant *DCK* expression increase, further enhanced by DAC exposure, no transfection-induced changes were found at the global DNA methylation level or in cell viability. In parallel, an integrative approach was applied to decipher DAC-induced, methylation-mediated, transcriptomic reprogramming. Besides large-scale hypomethylation, accompanied by up-regulation of gene expression across the entire genome, DAC also induced hypermethylation and down-regulation of numerous genes in both cell lines. Interestingly, *TET1* and *TET2* expression halved in JIMT-1 cells after DAC exposure, while DNMTs’ changes were not significant. The protein digestion and absorption pathway, containing numerous collagen and solute carrier genes, ranking second among membrane transport proteins, was the top enriched pathway in both cell lines when hypomethylated and up-regulated genes were considered. Moreover, the calcium signaling pathway, playing a significant role in drug resistance, was among the top enriched in JIMT-1 cells. Although low-dose DAC demonstrated its ability to normalize the expression of tumor suppressors, several oncogenes were also up-regulated, a finding, that supports previously raised concerns regarding its broad reprogramming potential. Importantly, our research provides evidence about the involvement of active demethylation in DAC-mediated transcriptional reprogramming.

## 1 Introduction

Breast cancer (BC) is the most common cancer diagnosed among women. Clinical management of patients involves multidisciplinary strategies combining surgery, radiotherapy, neoadjuvant, adjuvant, endocrine, or targeted therapies (Chew, 2001). However, about 30% of patients with early-stage BC experience disease recurrence due to the accumulation of molecular changes in tumor tissue (Colleoni et al., 2016). Besides well-studied genetic changes, less understood epigenetic alterations contribute to the development of drug resistance (Dworkin et al., 2009). Therefore, a considerable effort focuses on involving epigenetic inhibitors in the treatment of solid tumors, where evidence from preclinical studies demonstrates that epigenetic drugs (epi-drugs) can reverse aberrant expression patterns and sensitize resistant cancer cells to other forms of therapy. Although the efficacy of epi-drugs has shown promising results in hematological malignancies resulting in several regulatory authorities’ approvals, no epigenetic therapy, except for tazemetostat (Tazverik^®^), has been approved for solid tumors to date.

As aberrant epigenetic regulation was also demonstrated in BC, several clinical studies investigated the efficacy of DNA methyltransferase (DNMT) and histone deacetylase (HDAC) inhibitors used as monotherapies, showing their limited antitumor efficacy at the maximum tolerated doses (Buocikova et al., 2020). This finding was not surprising since solid tumors are considered more epigenetically complex and exhibit abnormal vascularization, specific tumor microenvironment, and more differentiated cells, leading to their decreased ability for epigenetic reprogramming (Ramachandran et al., 2015; Morel et al., 2020). Also, combinations of epi-drugs with cytotoxic drugs, targeted and hormone therapy, immunotherapy, radiotherapy, or other epi-drugs were assessed in BC with varying success (Falahi et al., 2014; Buocikova et al., 2020; Brown et al., 2022). Despite promising preclinical findings, results of clinical trials combining DNMT inhibitors 5-Aza-2′-deoxycytidine (decitabine, DAC) or azacytidine with panobinostat, tamoxifen, paclitaxel, carboplatin, doxorubicin, nab-paclitaxel, entinostat, fulvestrant, or durvalumab have been disappointing, due to limited efficacy and systemic toxicities (Buocikova et al., 2020). DAC combination with chemotherapy has also been studied in various BC subtypes (Hurtubise and Momparler, 2004; Mirza et al., 2010; Ari et al., 2011). In ER-negative BCs, HDAC inhibitors (entinostat, valproic acid, TSA) and azacytitine-entinostat combination have shown a high estrogen receptor (ER) re-expression and efficient restoration of sensitivity to antiestrogen treatment (Jang et al., 2004; Fortunati et al., 2010; Sabnis et al., 2011; Connolly et al., 2017). Recently a widespread effect of DAC on DNA methylation, including de-compaction of higher-order chromatin structure, has been reported in ER-positive endocrine-resistant BC patient-derived xenograft (PDX) model (Achinger-Kawecka et al., 2021). The authors showed that key enhancer ER binding sites were demethylated and reactivated after DAC treatment, resulting in the activation of tumor-suppressor gene (TSG) pathways.

These studies reveal a need for a better understanding of DAC mode of action, assessing its synergy with other anticancer agents, and identifying appropriate biomarkers for responders to this type of therapy (Yu et al., 2018; Morel et al., 2020). DAC is considered a pro-drug, converted to its active form by deoxycytidine kinase (DCK) or metabolized to an inactive form by cytidine deaminase (CDA) after the intracellular uptake. These pyrimidine metabolism enzymes play a crucial role in DAC sensitivity. CDA expression contributes to DAC resistance, converting DAC into uridine counterparts that do not deplete DNA methyltransferase 1 (DNMT1) (Qin et al., 2011; Gu et al., 2021). DAC incorporated into the DNA of proliferating cells forms a covalent bond with cysteine residues in the DNMT active sites and blocks the methylation of nascent DNA (Yang et al., 2010; Gnyszka et al., 2013). Its therapeutic features refer to its ability to facilitate promoter demethylation and reactivate silenced TSGs. Furthermore, the DAC-DNMT complex can initiate the DNA damage response, involving double-stranded DNA breaks that can result in growth inhibition and cell death (Seelan et al., 2018). The hypomethylating effect of DAC is pronounced at low concentrations, where the formation of DAC-DNMT adducts is limited, whereas, at higher concentrations, the formation of adducts leads to the cytotoxic effect (Qin et al., 2009). In agreement, high DAC doses induce significantly lower DNA hypomethylation compared to hundred-fold lower concentrations. This U-shaped hypomethylation dose-response curve of DAC treatment was reported in various cultured cells (Turchinovich et al., 2019). Surprisingly, several studies also reported DNMT inhibitors-mediated hypermethylation in specific cell types, including BC cells (Kastl et al., 2010; Chowdhury et al., 2015; Giri and Aittokallio, 2019). Therefore, it is necessary to dissect methylation- and toxicity-induced transcriptomic changes which could influence cells’ sensitivity to these compounds.

Recently we have shown that low-dose DAC increased the susceptibility of HER2-positive, trastuzumab-resistant JIMT-1 cell line-derived tumor xenografts to the cytotoxic drug doxorubicin (Buocikova et al., 2022). However, despite the high potency of this combination *in vitro*, synergy was not achieved *in vivo*. Therefore, we hypothesized that intracellular overexpression of DCK, one of the key enzymes involved in DAC metabolism, can potentiate DAC impact on cell methylome and thus increase its therapeutic efficacy. Besides testing this hypothesis, we used an integrated approach to decipher the effect of low-dose DAC on the methylome and transcriptome of the studied BC cell lines, representing distinct molecular subtypes.

## 2 Materials and methods

### 2.1 Cell cultures

Two human epithelial BC cell lines, authenticated in July 2018 by the short tandem repeat DNA profiling, were used in this study. JIMT-1 (DSMZ no.: ACC 589) is a trastuzumab-resistant cell line derived from high-grade invasive ductal carcinoma (ER-; progesterone receptor (PR)-; HER2+), while T-47D cells (ATCC® HTB-133™) represent luminal A subtype (ER+, PR+/-, and HER2-). Cells were cultivated at 37°C in a humidified atmosphere and 5% CO_2_ in high-glucose (4.5 g/l) Dulbecco’s modified Eagle medium (DMEM, PAA Laboratories GmbH, Austria) supplemented with 10% fetal bovine serum (FBS, Biochrom AG, Germany), 10 μg/ml gentamicin (Sandoz, Germany) and 2 mM glutamine (PAA Laboratories GmbH).

### 2.2 Transient transfection of the cells

The full-length open reading frame (ORF) of the human *DCK* gene was subcloned using standard cloning techniques from human cDNA. Cloning oligonucleotide PCR primers were designed to be homologic to the human *DCK* gene (22 bp part of the gene containing start and stop codon) and also to contain specific sites for restriction enzymes (XhoI and NotI). The resulting PCR product with the ORF of the human *DCK* gene was then subcloned into the pCIneo expression vector (Promega Corporation, Madison, WI) named as pCIneoDCK. Resulting plasmid was and verified by sequencing to exclude clones with mutations. Human cDNA used as a template for this PCR was prepared in our laboratory from human prostatic tumor cell line PC3. A green fluorescent protein (GFP) expression vector pCIneoGFP was used to determine transfection efficiency as a mock plasmid (kindly provided by G. Margison and J. Libby, Paterson Institute for Cancer Research, Christie Hospital, Manchester). Twenty-four hours prior to transfection with pCIneoDCK and pCIneoGFP vectors, JIMT-1 and T-47D cells were seeded in 24-well plates at a density of 1.5 × 10^5^ cells/well and 2 × 10^5^ cells/well, respectively. All transfections were carried out using 4 μl of FuGENE® HD Transfection Reagent (Promega Corporation) per 1 μg of plasmid DNA according to the manufacturer’s recommendations. After 24 h, the transfection medium was replaced with the fresh cultivation medium containing the selection reagent G418. Transfected cells were selected for 2 weeks with 1 mg/ml G418 (Sigma-Aldrich, Germany) for JIMT-1 and 0.5 mg/ml G418 for T-47D based on the previous G418 concentration testing. Schematic maps of the vectors pCIneoDCK and pCIneoGFP are presented in Supplementary Figure S1.

### 2.3 DCK sequencing

Sanger sequencing was used to control for *DCK* mutations. The cDNA of the DCK gene (ENSG00000156136) was transcribed from mRNA isolated from both cell lines using RevertAid First Strand cDNA Synthesis Kit (Thermo Fisher Scientific, United States) according to the manufacturer’s recommendations. cDNAs were amplified using HOT FIREPol® DNA Polymerase (Solis BioDyne, Estonia) with 10 μM primers (cDNA_DCK-F AAA​GTC​AAA​CCC​CGA​CAC​C; cDNA_DCK-R GCTGAAGTATCTGGAACCATTTG) with the PCR cycle set for an initial 15 min, denaturation at 95°C, followed by 30 cycles of denaturation at 95°C for 40 s, annelation at 60°C for 30 s, and polymerization at 72°C for 60 s, ended by 20 min of polymerization at 72°C. Samples were subsequently sequenced using the BigDye™ Terminator v3.1 Cycle Sequencing Kit according to the manufacturer’s recommendation and ABI Prism 3130xl Genetic Analyzer (Life Technologies, United States). Data analysis was performed by Sequencing Analysis Software v5.3 (Life Technologies). Sequences were aligned to the reference sequence (ENST00000286648.10) in ChromasPro Software v1.6 (Technelysium Pty Ltd, Australia).

### 2.4 Cell exposure

For cell viability analyses, 4.0 × 10^3^ cells/well of parental and transfected JIMT-1 and 5.0 × 10^3^ cells/well of parental and transfected T-47D cells were seeded into 96-well plates and treated every 24 h for a total of 72 h with different concentrations of DAC (MedChem Express, China) (0.05–100 µM). For the DNA damage, global DNA methylation, and whole-genome analyses, cells were seeded on Petri dishes (60 mm) at a density of 300 × 10^3^ cells/dish and treated with DAC every 24 h for a total of 72 h. The concentrations of DAC for DNA methylation assessment ranged between 0.05–50 µM, while for the whole-genome analyses, concentrations 1 µM for JIMT-1 and 4 µM for T-47D were selected based on prior viability testing. Afterward, the cells were pelleted for molecular analyses.

### 2.5 Cell viability

CellTiter-Glo® Luminescent Cell Viability Assay (Promega Corporation) was used to establish cell viability which was evaluated by GloMax® Discover Microplate Reader (Promega Corporation). Cell viability was determined as the luminescence intensity relative to untreated control cells (set to 100%), and the results are presented as means ± standard errors (SEM) from three independent experiments in quadruplicates.

### 2.6 Genotoxic effects

DNA strand breaks (SB) induction by DAC exposure was evaluated by comet assay as published previously (Buocikova et al., 2022). Briefly, exposed cells were embedded in low-melting-point (LMP 0.8%, Sigma-Aldrich) agarose, placed on microscope slides (200–500 cells per gel), and incubated for 1 h in lysis solution (2.5 M NaCl, 0.1 M EDTA, 10 mM Tris, 10% v/v Triton X-100, pH 10, 4 °C). Then, slides were transferred to an electrophoresis box and immersed in an alkaline solution (0.3 M NaOH, 1 mM EDTA, pH > 13, 4 °C). After 20 min unwinding time, a 25 V (1.25 V/cm, Consort EV202) voltage was applied for 20 min at 4 °C. The slides were washed in PBS and H_2_O and left to dry. Before scoring, slides were stained with SYBR gold (Sigma-Aldrich) and examined with a fluorescence microscope (DMI 6000 B, Leica Microsystems, Germany) equipped with an SYBR photographic filter (Thermo Fischer Scientific), using the computerized image analysis Comet Assay IV 4.3.1 software (Perceptive Instruments, UK). The median percentage of DNA in the tail (% of tail DNA) was used for DNA damage measurement. One hundred comets were scored per treatment group.

As a positive control, cells embedded in gel on the slide were exposed to 100 µM H_2_O_2_ (Sigma-Aldrich). These data are reported in Supplementary Figure S2. The Alamar Blue assay was used in parallel with the comet assay as a standard procedure, providing an overview of tested drug cytotoxicity, which might affect the results (Buocikova et al., 2022).

### 2.7 Flow cytometry

Annexin V Apoptosis Detection Kit APC (eBioscience, Thermo Fisher Scientific) was used to evaluate the percentage of apoptotic, necrotic, and viable cells, according to the manufacturer’s recommendations. Cells in density 100 × 10^3^ cells/well were seeded on 6-well plates, and after that, DAC was added every 24 h, in a total of 72 h, in the concentration of 1 µM for parental and transfected JIMT-1 and 4 µM for parental and transfected T-47D cells. Harvested cells were washed with Annexin V binding buffer and incubated for 20 min with APC-conjugated Annexin V at room temperature, protected from light. Fluorescent DNA binding dye 7-Amino-Actinomycin D (7AAD, 2 µg/ml) was used for the detection of dead cells. Analysis was performed on BD FACS Canto™ II flow cytometer, and data were analyzed with the FCS Express program; values represent means of triplicates ± SEM.

### 2.8 RNA extraction and RT-PCR

RNeasy Mini Kit (Qiagen, Germany) was used to isolate total RNA from cell pellets, using the On-column DNase digestion step with the RNase-Free DNase Set (Qiagen). Extracted RNA quality and quantity were evaluated using NanoDrop® ND-1000 spectrophotometer (Thermo Fisher Scientific). A total of 4 µg of RNA from each sample was reverse transcribed using a Revert Aid^TM^ H Minus First Strand cDNA Synthesis Kit (Thermo Fisher Scientific). DCK expression was analyzed with individually designed primers (F 5′-TCT​GAG​GGG​ACC​CGC​ATC​AA-3′ and R 5′-TGC​ACC​ATC​TGG​CAA​CAG​GTT-3'; product length 133 bp) using *HPRT1* (F 5′-GGA​CTA​ATT​ATG​GAC​AGG​ACT-3′ and R 5′-GCT​CTT​CAG​TCT​GAT​AAA​ATC​TAC-3'; product length 194 bp), for normalization. The reaction mixture contained 7.5 µl of 2× GoTaq® qPCR Master Mix (Promega), 1 µl (0.67 μM) of each forward and reverse primer, 4.5 µl ultrapure DNase/RNase-free water, and 50 ng cDNA. Amplification was carried out in triplicates on a Bio-Rad CFX96 real-time PCR detection system (Bio-Rad, United States). TaqMan gene expression assays for *CDA* (Hs00156401_m1), *TET1* (Hs00286756_m1), *DNMT1* (Hs00945875_m1), *DNMT3A* (Hs01027166_m1), and *DNMT3B* (Hs00171876_m1) genes were employed to analyze their relative mRNA expression, using *HPRT1* (Hs02800695_m1) for normalization. The reaction mixture contained 10 µl of 2x Taq-Man gene expression master mix (ThermoFisher Scientific), 1 µl of 20x TaqMan® Assay, and 9 µl of cDNA template (50 ng) and ultrapure DNase/RNase-free water. The quantitative PCR (qPCR) was performed on a Bio-Rad CFX96 real-time PCR detection system (Bio-Rad) in triplicates. The cycling conditions were as follows: hold 50 °C 2 min for UNG incubation, hold 95 °C 10 min for polymerase activation, and 40 cycles denaturation at 95 °C for 15 s followed by annealing/extension step 60 °C for 60 s. Obtained Cq values were used to quantify relative gene expression changes by employing the relative expression software tool REST (REST 2009-RG Mode, Qiagen) developed by Pfaffl et al. (Pfaffl et al., 2002).

### 2.9 Pyrosequencing

Genomic DNA (2 µg) from studied cell lines was used for sodium bisulfite treatment using the EpiTect Bisulfite kit (Qiagen). This approach allows complete conversion of unmethylated cytosines to uracils while methylated cytosines remain unaffected, which enables the detection of methylated CpGs. DNA methylation of the long-interspersed nucleotide element 1 (LINE-1) was evaluated by the quantitative pyrosequencing method. The PCR amplification was performed using PyroMark PCR Kit (Qiagen) following the manufacturer’s instructions. Global DNA methylation analysis was done with the PyroMark Q24 CpG LINE-1 kit (Qiagen). DNA methylation of three CpG sites in positions 331 to 318 of the LINE-1 sequence (GenBank accession number X58075) was evaluated. Pyrosequencing was carried out using PyroMark Gold Q24 Reagents (Qiagen) on a PyroMark Q24 platform. Data analysis was performed by PyroMark Q24 2.0.6. software (Qiagen).

### 2.10 Western blot

Proteins were isolated from tumor cell lines using RIPA lysis buffer (Cell Signaling Technology, United States) supplemented with Complete™ Protease Inhibitor Cocktail, as recommended by the manufacturer (Roche, Germany). Total protein concentration was quantified by Pierce™ BCA Protein Assay Kit (Thermo Fisher Scientific). Protein samples were diluted in 4x Laemmli Sample Buffer (Bio-Rad) and denatured for 8 min at 96°C prior to use. 30 µg of proteins were separated by 12% SDS-PAGE and transferred by the Trans-Blot Turbo to the Nitrocellulose Membrane 0.45 μm (Thermo Fisher Scientific). Membranes were blocked in 5% w/v non-fat dry milk (Artifex Instant s. r.o., Czech Republic) in TBS (20 mM Tris, 150 mM NaCl) and incubated with DCK Rabbit mAb (1:5,000) (cat. no. PA5-27787), CDA Rabbit mAb (1:5,000) (cat. no. PA5-84630) (both Thermo Fisher Scientific) and anti-β-actin mouse monoclonal (1:4,000) primary antibodies (Sigma-Aldrich, cat. no. A1978). Specific binding of the antibodies was detected with appropriate secondary antibodies Goat anti-Rabbit IgG (H + L) Highly Cross-Adsorbed Secondary Antibody, Alexa Fluor 680 (1:10,000) (cat. no. A-21058), and Alexa Fluor 790 (1:10,000) (cat. no. A11375) and Goat anti-Mouse IgG (H + L) Highly Cross-Adsorbed Secondary Antibody, Alexa Fluor 680 (1:10,000) (cat. no. A32729) (Thermo Fisher Scientific). Membranes were visualized using Odyssey® Fc (LI-COR) imaging system. Levels of detected proteins were quantified by densitometry, using ImageJ/Fiji software, and expressed as protein/loading control ratio relative to vehicle control.

### 2.11 Statistical analysis

Normality of distribution was tested using Shapiro–Wilk test. Significant differences between normally distributed data were assessed by Student t-test or one-way analysis of variance (ANOVA) and appropriate post-hoc tests depending on assumed variances. Non-normally distributed data were evaluated using Mann-Whitney U-test or Kruskal–Wallis test, followed by Dunn or Dunn–Bonferroni post hoc methods. Data were analyzed using SPSS software package version 23 (IBM SPSS, Inc., United States). Differences with *p* < 0.05 were considered to be statistically significant.

### 2.12 Whole transcriptome and whole-genome methylation screening experiments

All whole-genome analyses were done as published previously (Buocikova et al., 2022). Three independent experiments comparing DAC-exposed cells against controls were evaluated.

#### 2.12.1 RNA-seq analysis

For RNA-seq analysis, RNA was extracted using the RNeasy Mini Kit (Qiagen). The quality of isolated RNA was analyzed using the Agilent RNA 6000 Nano Kit (Agilent Technologies, United States), and only RNA samples having RIN > 7.5 and 28S/18S ratio ≥ 0.8 were selected for RNA-seq analysis. Whole transcriptome expression analysis was done using the BGI DNBseq PE100 RNA-seq platform. For data analysis workflow, STAR (Dobin et al., 2013), HTSeq (Anders et al., 2015), and DESeq2 (Love et al., 2014) algorithms were applied. STAR aligner was used to map the reads to the Hg38 reference genome, while HTSeq-count was used for gene quantification and generation of gene expression counts. Differential gene expression analysis was implemented using DESeq2.

#### 2.12.2 Methylation analysis

DNA extraction from cell pellets was performed with a QIAmp DNA Mini kit (Qiagen), following the manufacturer’s instructions. NanoDrop® ND-1000 spectrophotometer (Thermo Fisher Scientific) was used to control for extracted DNA quantity and quality. The Human Infinium Methylation EPIC Bead Chip array, measuring 850,000 methylation sites, was used according to the manufacturer’s instructions to assess differentially methylated CpGs. Arrays were processed *via* a pipeline, including the R ChAMP package (Tian et al., 2017). Data were normalized through a BMIQ (beta-mixture quantile) procedure and corrected for batch effect through the ComBat algorithm (Johnson et al., 2007). Individual methylation value (β value) was evaluated for each CpG site, ranking from 0 for unmethylated to 1 for fully methylated CpGs, and batch-corrected β values were subjected to paired differential methylation analysis in the ChAMP package.

#### 2.12.3 Integration of whole-genome methylation and transcription data

Differential gene expression analysis was implemented using DESeq2, with abs(log2FoldChange) ≥ 1.0 and false discovery rate (FDR) adjusted *p*-value < 0.05 cut-offs, while *p*-value ≤ 0.01 was set as the significance threshold for differentially methylated probes, based on β values analyzed using ChAMP. In an integrative analysis, determining the intersection between the differentially methylated and expressed genes, the activation or inactivation of TSGs and oncogenic events were characterized using TSGene 2.0 and ONGene databases (Zhao et al., 2016; Liu et al., 2017).

## 3 Results

**FIGURE 1:**
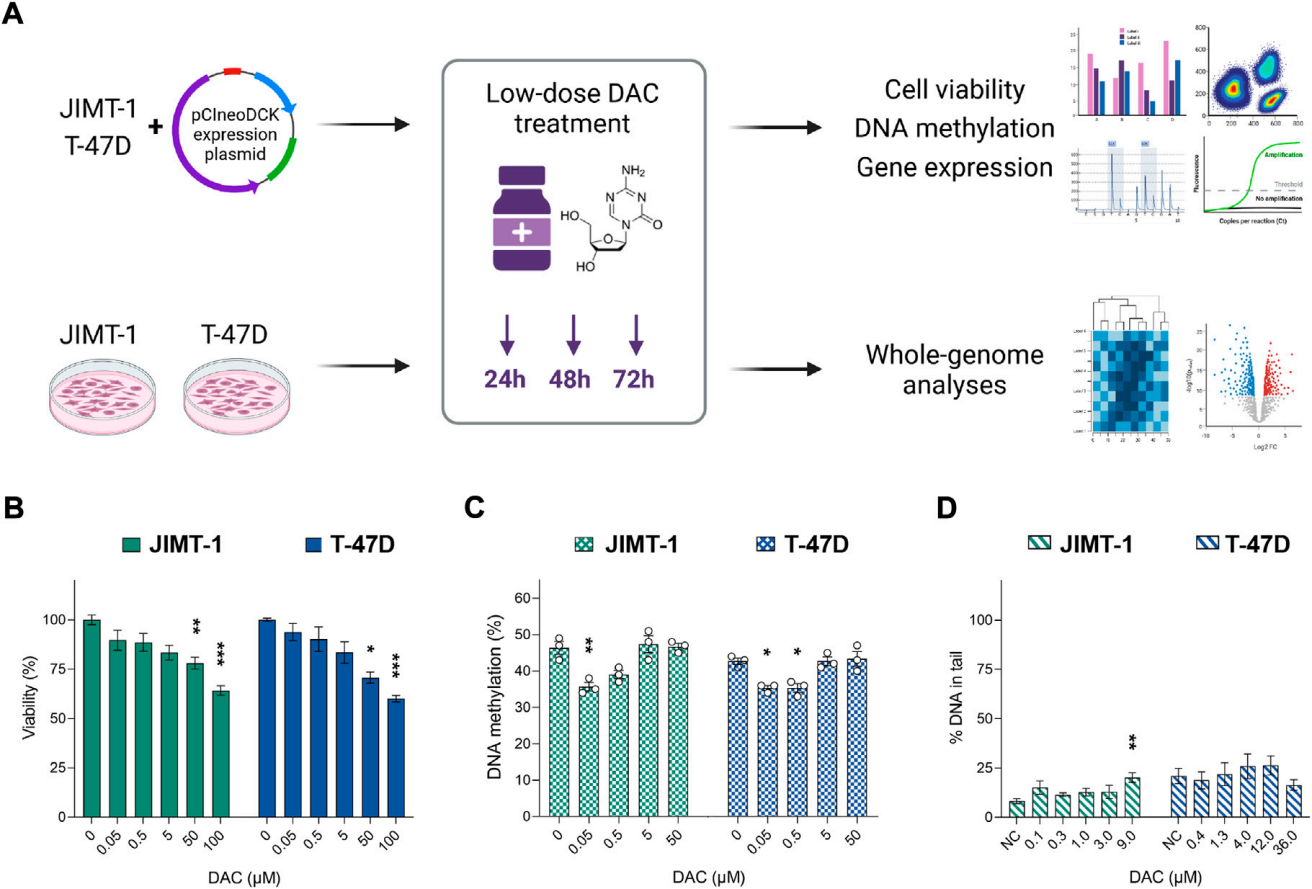
The impact of increasing decitabine (DAC) concentrations on cell viability, DNA methylation, and DNA damage. **(A)** Experimental design; **(B)** Cell viability changes induced by exposure to increasing DAC concentrations added every 24 h for a total of 72 h. Relative cell viability values were evaluated by luminescent viability assay and expressed as mean ± SEM. Differences to untreated cells; *p < 0.05, **p < 0.01, ***p < 0.001; **(C)** Exposure-induced changes in global DNA methylation levels; **(D)** Impact of DAC exposure on DNA damage, measured as strand breaks by comet assay.

The main aim of this study was to assess the impact of DCK overexpression on DAC efficacy and to investigate the effect of low-dose DAC on JIMT-1 and T-47D BC cells’ methylome and transcriptome (Figure 1A).

### 3.1 Impact of DAC exposure on cell viability, global DNA methylation, and DNA damage

Initially, we examined the effect of several DAC concentrations (0.05–100 µM) on cell viability (Figure 1B) and global DNA methylation changes (Figure 1C). A concentration-dependent reduction in cell viability reached significance at concentrations over 50 µM in both cell lines. However, a decrease in global DNA methylation was detected only at concentrations ranging between 0.05 and 0.5 µM. At concentrations higher than 5 µM, the methylation status was comparable with the control cells. To avoid DAC dual mode of action, specifically cell toxicity associated with DNA damage, non-cytotoxic concentrations of DAC (approximately IC_20_) were selected for further experiments (1 µM for more sensitive JIMT-1 and 4 µM for T-47D cells). Due to low stability and cell division-dependent effect, DAC was administered repeatedly, every 24 h for a total of 72 h.

The comet assay method was used to evaluate genotoxic effect of DAC. No DAC-induced increase in SB was found, except for the highest concentration in JIMT-1 cells, where cell viability was as low as 31.8 ± 3.9% as measured by the Alamar Blue assay (Figure 1D, Supplementary Figure S2). Thus, it cannot be excluded that the DNA damage at this concentration was secondary due to the cytotoxic effect. However, 1 µM concentration was used in JIMT-1 cells for further analyses.

### 3.2 The effect of intracellular DCK overexpression

Given that DAC is a pro-drug, metabolized to its active form by the DCK enzyme, we hypothesized that an intracellular increase in *DCK* expression might improve the efficacy of DAC treatment. The absence of chromosomal mutations in *DCK* gene was confirmed by Sanger sequencing in both cell lines.

#### 3.2.1 Cell transfection with pCIneoDCK expression vector

**FIGURE 2:**
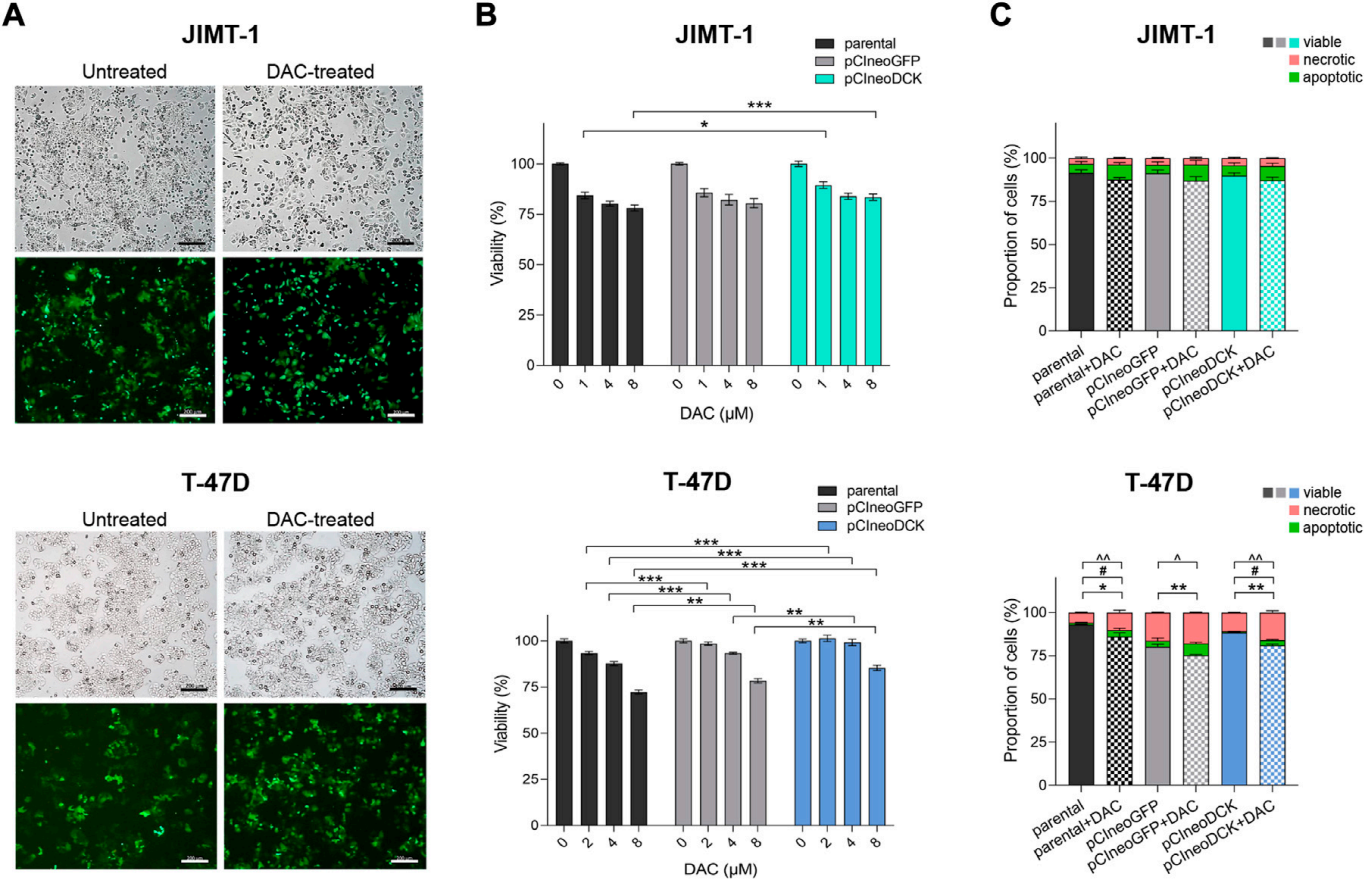
Effect of cell transfection on cell survival and response to decitabine (DAC). **(A)** The morphology and evaluation of transfection efficiency in JIMT-1 and T-47D cell lines transfected with GFP expression vector pCIneoGFP (mock plasmid). Representative images of untreated and DAC-treated pCIneoGFP cells were taken on a light microscope Axio Vert. A1 ZEISS, magnification 5 x; **(B)** Cell viability assessment of parental and transfected JIMT-1and T-47D cells after DAC treatment, added every 24 h for a total of 72 h. Relative cell viability values were evaluated by luminescent viability assay and expressed as mean ± SEM; **(C)** Flow cytometry measurement of the apoptosis and necrosis in parental and transfected JIMT-1 and T-47D cells. Statistically significant difference from negative control according to one-way ANOVA (Bonferroni’s or Tamhane’s multiple comparisons test), *p < 0.05, **p < 0.01, ***p < 0.001 or Kruskal-Wallis test; ^ viable, #apoptotic, *necrotic cells.

To increase intracellular DCK expression, JIMT-1 and T-47D cells were transiently transfected with pCIneoDCK expression plasmid (Supplementary Figure S1A). The pCIneoGFP vector was used as a mock transfection control to evaluate the transfection efficiency, reaching 50% after selection with antibiotic G418 (Supplementary Figure S1B, Figure 2A). In JIMT-1 cells (Figures 2B,C), no effect of pCIneoGFP or pCIneoDCK plasmids on cell viability was found in parental or DAC-exposed cells, and the exposure did not induce apoptosis or necrosis after 72-hour treatment. However, transfection with both plasmids slightly increased the proliferation of T-47D cells, which was accompanied by DAC exposure-induced apoptosis and necrosis (Figure 2C).

#### 3.2.2 Gene and protein expression changes, global DNA methylation

**FIGURE 3:**
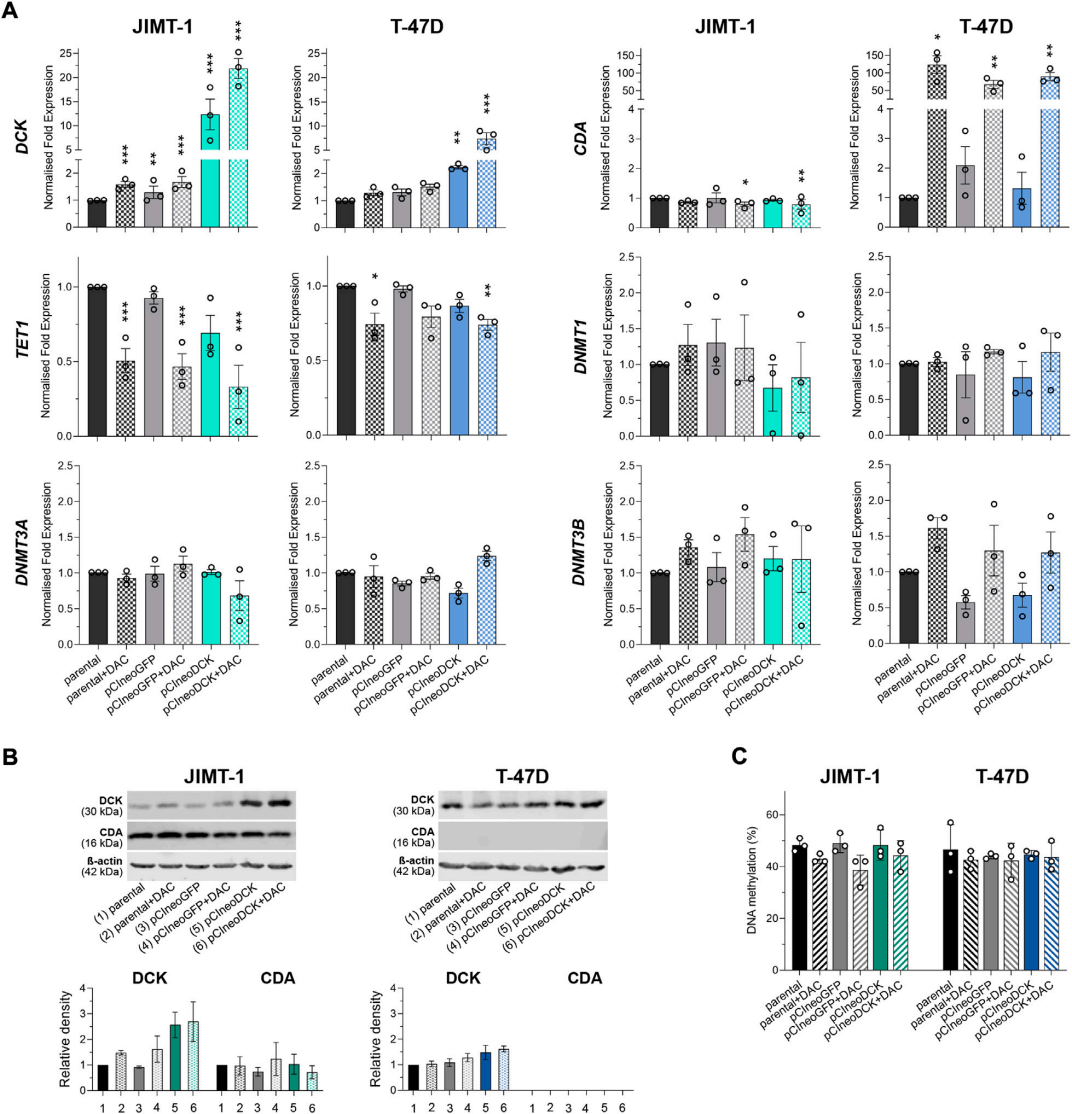
Gene and protein expression of enzymes involved in pyrimidine metabolism after transient transfection with DCK plasmid. **(A)** *DCK*, *CDA*, *TET1*, *DNMT1*, *DNMT3A*, and *DNMT3B* gene expression changes in JIMT-1 and T-47D cells due to pCIneoDCK transfection and DAC exposure. Mean values from three independent experiments are expressed as normalized gene expression compared to parental cells; error bars represent SEM; *p < 0.05, **p < 0.01, ***p < 0.001; **(B)** Representative results of Western blot analysis for DCK and CDA expression after individual treatments and its quantification by densitometry from three experiments; **(C)** LINE-1 DNA methylation in parental and transfected JIMT-1 and T-47D cells, evaluated by pyrosequencing. DNA methylation values (%) are presented as mean ± SEM. No significant differences were found.

The transient transfection with pCIneoDCK vector caused a significant increase in *DCK* expression in both cell lines but more efficiently in JIMT-1 compared to untreated parental cells (JIMT-1/pCIneoDCK vs. parental cells fold change (FC) = 11.2, *p* < 0.001; T-47D/pCIneoDCK, FC = 2.3, *p* = 0.003) (Figures 3A,B). This effect was more pronounced by exposure to DAC (JIMT-1/pCIneoDCK + DAC, FC = 21.6, *p* < 0.001; T-47D/pCIneoDCK + DAC, FC = 7.2, *p* < 0.001). Interestingly, although DAC-induced up-regulation of *CDA* mRNA in parental and transfected T-47D cells (parental + DAC, FC = 126.3, *p* = 0.013; pCIneoGFP + DAC, FC = 110.4, *p* = 0.004; pCIneoDCK + DAC, FC = 145.2, *p* = 0.001), this up-regulation was not confirmed by western blot (Figures 3A,B). In the JIMT-1 cell line, no exposure-induced changes in *CDA* expression were identified. In parallel, DAC caused down-regulation of *TET1* gene, which was more apparent in JIMT-1 cells (FC = 0.50, *p* < 0.001 in DAC exposed parental cells; FC = 0.47 in pCIneoGFP + DAC, *p* < 0.001 and FC = 0.26, *p* < 0.001 in pCIneoDCK + DAC vs. untreated parental cells). Changes in DNMTs gene expression did not reach the cut-off (FC ≥ 2, equivalent to abs (log2FC) ≥ 1). DCK overexpression did not significantly influence global DNA methylation measured by pyrosequencing as LINE-1 DNA methylation (Figure 3C).

### 3.3 Methylomic changes induced by exposure to low-doses DAC

**FIGURE 4:**
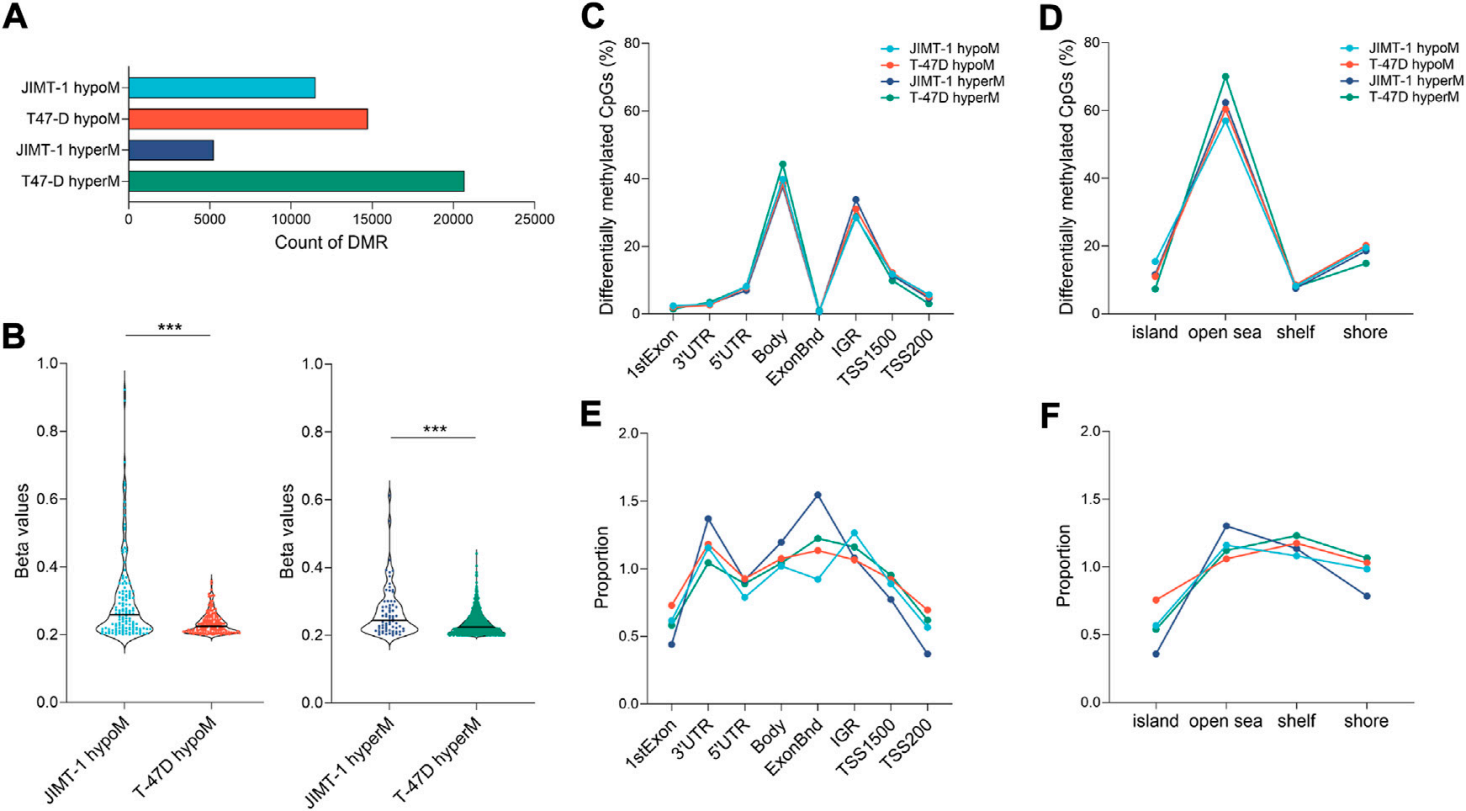
Extend of DNA methylation changes in studied cell lines. **(A)** The number of hypo- and hypermethylated CpGs in JIMT-1 and T-47D cell lines (*p*-value ≤ 0.01); **(B)** Violin plots showing spread out the ∆β beta values (≥ 0.2) for differentially hypo- and hypermethylated probes (*p*-value ≤ 0.01) identified between treated and control JIMT-1 and T-47D cells, ***p < 0.001; Distribution of DAC-induced DNA methylation changes (*p*-value ≤ 0.01) across the **(C)** functional and **(D)** regulatory regions in the genome and **(E, F)** their normalization to the number of analyzed CpGs, respectively; Abbreviations: hyperM—hypermethylated, hypoM—hypomethylated, Bnd-boundaries, IGR-intergenic regions, TSS-transcription start site.

To study the extent of low-dose DAC on epigenomic reprogramming, we analyzed genome-wide DNA methylation data obtained through the Infinium EPIC Methylation platform. DAC exposure induced hypo- and hypermethylation in both cell lines (Figure 4A). At equitoxic concentrations, JIMT-1 cells were hypomethylated less frequently (11503 probes) than T-47D cells (14737 probes). Hypermethylation occurred in 5,264 CpGs only in the JIMT-1 cell line, in contrast to 20684 CpGs in T-47D cells. Similar differences were found when Δβ (cut-off ≥ 0.2) was considered. However, as shown in Figure 4B, the extent of Δβ values, particularly for DNA hypomethylation, was significantly higher in JIMT-1 than in T-47D cells (*p* < 0.001).

Only minor differences were found between cell lines when DNA methylation changes were assessed at individual genomic locations (Figures 4C,D). Interestingly, the frequency of these changes did not significantly differ between hypo- and hypermethylation. However, differences between cell lines became more pronounced when data were normalized against the number of analyzed CpGs at given locations (Figures 4E,F). The biggest differences were found at 3′UTR and exon boundaries, where hypomethylated CpGs were less frequent in JIMT-1 cells, while they were most frequently hypermethylated at the same positions. On the other hand, hypermethylated CpGs in the JIMT-1 cell line were less common in TSS1500 and TSS200, which was also reflected in the methylation proportion of CpG islands, which were more frequently hypomethylated in T-47D cells.

#### 3.3.1 Integration of DNA methylation and gene expression data

**FIGURE 5:**
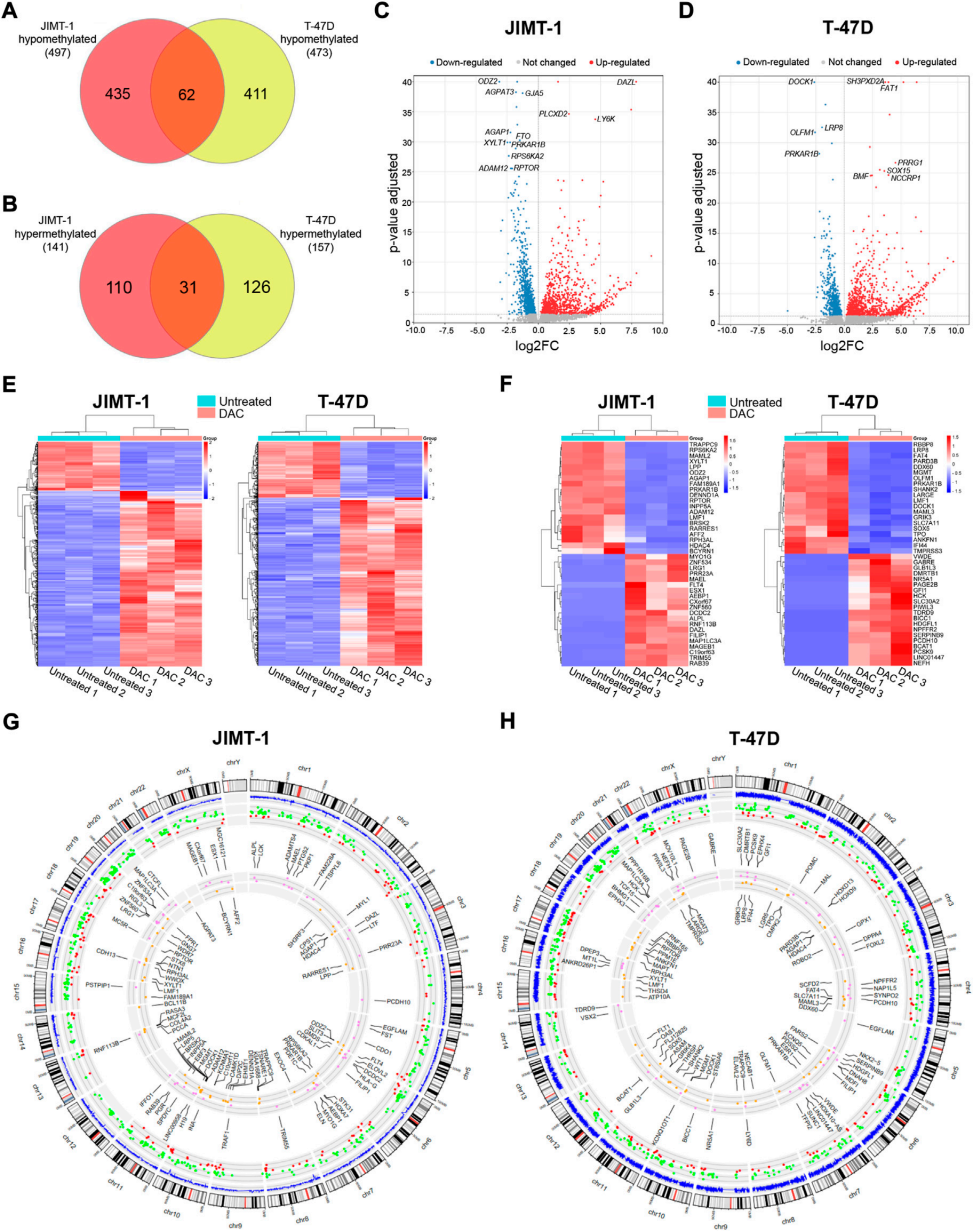
Integration of decitabine (DAC)-induced DNA methylation and gene expression changes. The number of **(A)** hypomethylated and **(B)** hypermethylated genes after DAC treatment in JIMT-1 and T-47D cell lines (*p*-value ≤ 0.01; abs(log2FC) ≥ 1, FDR *p*-value ≤ 0.5 set as cut-offs for DNA methylation and gene expression, respectively); Volcano scatter plots showing the distribution -log10 (*p*-value) (y-axis) and log2FC (x-axis) of methylation-mediated changes in mRNA expression induced by exposure to DAC compared to non-treated controls for **(C)** JIMT-1 and **(D)** T-47D cells. In each plot, significantly up-regulated entities are highlighted by red and down-regulated by blue. Non-significant findings are presented as grey dots; Heatmaps depict **(E)** all DAC-induced DNA methylation-mediated gene expression changes in JIMT-1 and T-47D cells, and **(F)** the top 20 differentially regulated genes, respectively; Circos graphs show the integration of methylation and gene expression changes in **(G)** JIMT-1 and **(H)** T-47D cells. The outer circle is the chromosome idiogram of the human genome (based on G-banding, centromere highlighted in red). Blue outer circos plots demonstrate the culmination of methylation data (*p* < 0.01). The second plot represents hypomethylated and up-regulated (green dots) and hypermethylated and down-regulated (red dots) genes. The inner plot shows the chromosomal location of the top 50 hypomethylated/up-regulated (violet) and hypermethylated/down-regulated (yellow) genes.

The results for DAC-induced changes on the transcriptomic level were published in detail previously (Buocikova et al., 2022). Integration of DNA methylation (*p*-value < 0.01) and gene expression changes with FDR < 0.05 and abs(log2FC) ≥ 1) cut-offs revealed that 497 genes were hypomethylated and up-regulated in JIMT-1 cells, and 473 genes in T-47D cells with 62 common genes for both cell lines (Figure 5A, Supplementary Table S1). On the contrary, 141 and 157 genes were hypermethylated and down-regulated, respectively, with 31 common genes (Figure 5B, Supplementary Table S1). The volcano plots demonstrate the extent of methylation-mediated deregulation of gene expression. In JIMT-1 cells, the top up-regulated genes were *DAZL*, *PLCXD2,* and *LY6K* (Figure 5C), while in the T-47D cell line, they were *SH3PXD2A*, *FAT1,* and *PRRG1* (Figure 5D). The heatmaps show all anticorrelated genes (Figure 5E) and the top 20 deregulated genes with log2FC ranging from 9.1 to 5.7 and -3.1 to -2.0 in JIMT-1 and from 9.6 to 6.5 and -5.0 to -2.0 in T-47D cells, respectively (Figure 5F). Among the top down-regulated genes in JIMT-1 cell line belong *HDAC4* (log2FC = -3.0) and the top gene in T-47D cell line was *SOX5* (log2FC = -5.0).

Importantly, 35 of hypomethylated and up-regulated genes were TSGs, including *CDO1* (log2FC = 5.5), *CXCL12* (log2FC = 4.9), *ACHE* (log2FC = 3.3), *ITGA7* (log2FC = 2.7) in JIMT-1 cell line and 51, among them *NGFR* (log2FC = 3.7), *RASSF2* (log2FC = 3.2), *ACVR1C* (log2FC = 3.2), *NR4A3* (log2FC = 2.8), *PPP1RB1B* (log2FC = 2.3), *PRICKLE1* (log2FC = 2.3) in T-47D cells. However, 25 proto-oncogenes and candidate oncogenes were also up-regulated, such as *RET* (log2FC = 4.7), *NTRK1* (log2FC = 4.6), *AQP1* (log2FC = 3.5), *CYP24A1* (log2FC = 2.4) or *ALK* (log2FC = 2.3) in JIMT-1 cells and 28, including *PPP1R14A* (log2FC = 4.6), *HOXA9* (log2FC = 4.2), *FES* (log2FC = 3.7), *TNFRSF1B* (log2FC = 2.5) and *MET* (log2FC = 2.0) in T-47D cells. We also found several hypermethylated and down-regulated TSGs in both cell lines, 10 in JIMT-1 and 21 in T-47D cells, with log2FC below -2, except for *MAML2* (log2FC = -2.9). Notably, *ESR1* was down-regulated in T-47D cells (log2FC = -1.8) (Supplementary Table S1). All listed genes were involved in deregulated signaling pathways.

Circos graphs present the distribution of differentially methylated CpG dinucleotides (*p* ≤ 0.01) across the genome, including anticorrelations and 50 top deregulated genes in JIMT-1 (Figure 5G) and T-47D (Figure 5H) cell lines. The distribution of hypo- and hypermethylation (Supplementary Table S1) demonstrates a homogenous pattern across the genome, depending on the gene number located on individual chromosomes. DNA methylation-mediated transcriptomic changes were also evenly distributed.

**FIGURE 6:**
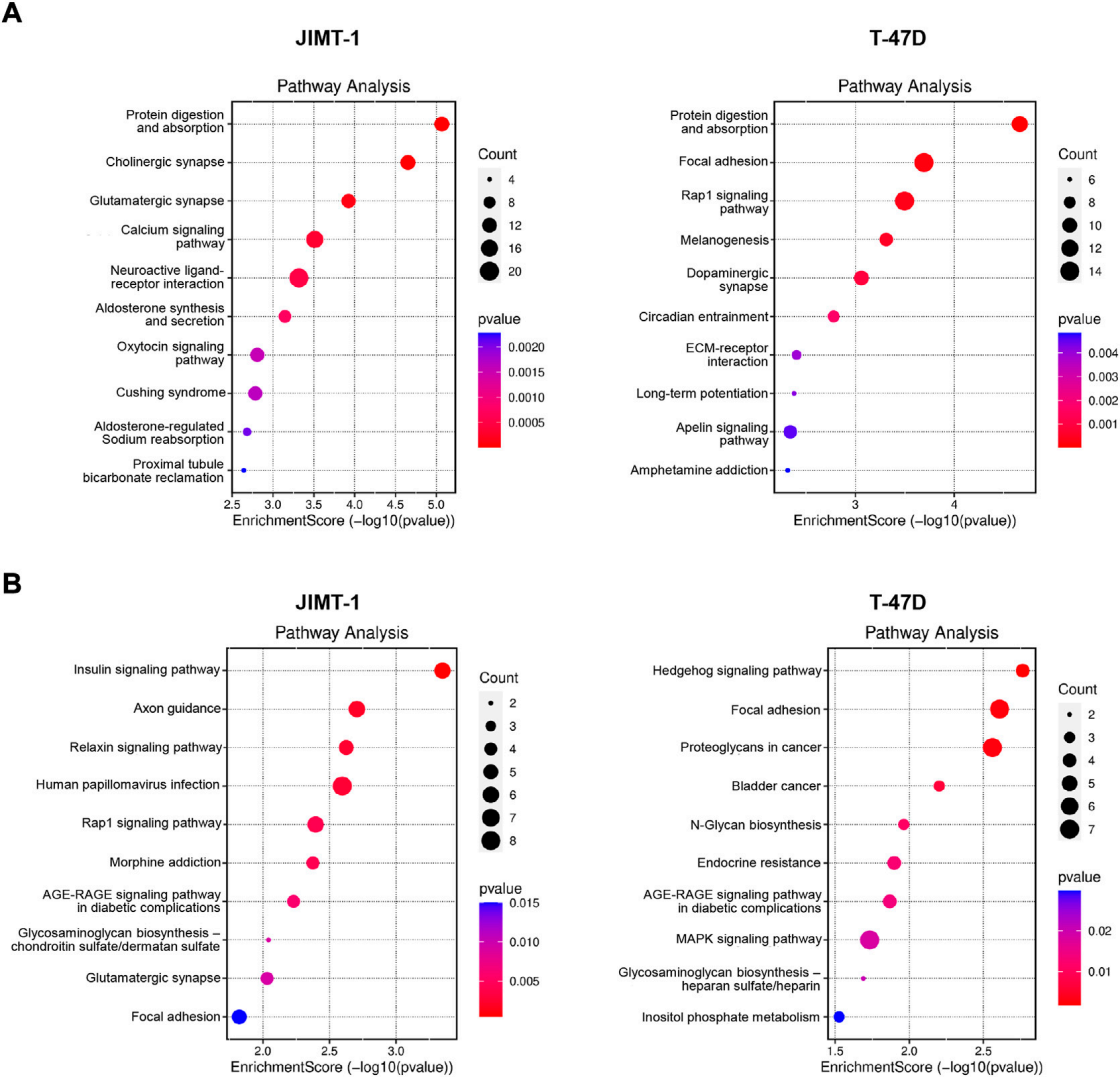
Pathway analysis for **(A)** significantly hypomethylated/up-regulated and **(B)** hypermethylated/down-regulated genes in JIMT-1 and T-47D cells, respectively.

When hypomethylated genes were analyzed, among the top 10 deregulated pathways in JIMT-1 cells were protein digestion and absorption pathway, neuroactive ligand-receptor interaction pathway, and calcium signaling pathway (Figure 6A). Interestingly, protein digestion and absorption pathway were among the top enriched also in T-47D cells when hypomethylated genes were considered, suggesting a conserved biological circuit, followed by focal adhesion, melanogenesis, and ECM-receptor interaction (Figure 6A). Due to the relatively small number of hypermethylated and down-regulated genes, the number of enriched pathways was limited with few involved genes (Figure 6B). All pathway data are available in Supplementary Table S2.

## 4 Discussion

An increasing body of evidence demonstrates that epigenome modulation could perpetrate a significant role in the treatment of resistant cancers, sensitizing them to other therapeutic approaches (Morel et al., 2020). The DCK-mediated phosphorylation is a rate-limiting step of the deoxynucleoside salvage pathway that plays a crucial role in activating several anticancer drugs, such as fludarabine, gemcitabine, and cladribine, including DAC (Klanova et al., 2014). Increased DCK expression found in several cancers, including poor prognosis BC, indicates that these patients might be susceptible to treatment with nucleoside analogs (Geutjes et al., 2012). Conversely, its impaired expression or activity leads to treatment resistance. This hypothesis is supported by the fact that *DCK* up-regulation induced by retroviral transduction with the DCK vector was able to sensitize non-malignant MCF 10A and malignant HCC1954 BC cells to DAC and gemcitabine (Geutjes et al., 2012). Moreover, Qin et al., 2009 demonstrated that transfection of wild-type *DCK* gene restored DAC sensitivity in the HL60 leukemia cells, resistant to DAC treatment due to homozygous *DCK* mutation. Despite these promising results, we did not observe any decrease in cell viability or DNA methylation attributable to elevated intracellular DCK levels in JIMT-1 and T-47D cells. However, we are aware that the manifold, cell-specific character of the DAC mode of action renders this type of comparison difficult.

CDA, another critical enzyme involved in pyrimidine metabolism, rapidly catabolizes DAC into uridine counterparts (Zauri et al., 2015). Its overexpression has also been linked to resistance to cytidine analogs—such as cytosine arabinoside, 5-azacytidine, or gemcitabine. DAC-induced *DCK* and *CDA* gene expression changes differed markedly between studied cell lines. While exposure to DAC up-regulated *CDA* mRNA expression in parental and transfected T-47D cells (log2FC = 5.9 by RNASeq), no effect on *CDA* expression was observed in the JIMT-1 cell line, having nearly 3,000 times higher expression of this gene (Buocikova et al., 2022). The lack of enhanced CDA at the protein level might indicate problems with the translation of mRNA into protein due to post-transcriptional regulation or limited protein expression in this cell line. However, our findings support evidence that pyrimidine metabolism consists of a complex network that senses and regulates amounts of deoxynucleotides in the cell. Importantly, continuous pro-drug exposure was shown to induce stabilized compensating adaptive response in the cells aiming to prevent DNMT1 depletion (Gu et al., 2021). Consistently with our findings, DAC had little or no effect on *CDA* transcript levels in cell lines having constitutively high *CDA* expression, while it substantially increased *CDA* mRNA levels in CDA-deficient cancer cell lines (Mameri et al., 2017).

Variability in DAC susceptibility could be affected by the genetic and epigenetic background of a particular cell line. At the same time, different genes and genomic regions may respond differently to DAC treatment. Accordingly, by applying whole-genome approaches, we found large-scale, cell-type-specific transcriptomic reprogramming, mediated by more subtle methylation changes. While the most significant differences between cell lines occurred in exon boundaries, DAC-induced changes were generally less frequent in the CpG islands, including the first exon, TSS200, and TSS1500, than in other genomic regions. These findings are consistent with previously published data, showing that non-CpG island regions, especially CpG sites located in repetitive sequences, were more vulnerable to demethylation. In contrast, demethylation-resistant CpGs were located in promoters associated with the binding of polycomb repressive complex 2 (Hagemann et al., 2011).

Our results denote new molecular DAC targets and pathways in studied BC cell lines. Among others, in trastuzumab-resistant JIMT-1 cells, DAC exposure inhibited the expression of *HDAC4,* histone deacetylase overexpressed in resistant BC tumors (Wang et al., 2014). On the contrary, in T-47D cells, low-dose DAC down-regulated *SOX5*, the transcription factor highly expressed in BC tissues, which is involved in BC proliferation and invasion and associated with a reduced overall survival (Sun et al., 2019). The top enriched protein digestion and absorption pathway, containing numerous collagen and the solute carrier (SLC) genes, ranks second among membrane transport proteins and plays a significant role in regulating cellular functions, primarily in tumor biology. The neuroactive ligand-receptor interaction pathway mainly consists of neuroreceptor genes and is involved in environmental information processing, signaling molecule interaction, as well as development and differentiation cues. The other enriched pathways are, to some extent, related to the neuroactive ligand receptors pathway, e. g. cholinergic synapse, glutamatergic synapse, aldosterone synthesis and secretion, and oxytocin signaling pathways. Ion channels are versatile regulators of several physiological and pathophysiological mechanisms, including cancer-relevant processes such as tumor progression, apoptosis inhibition, proliferation, migration, invasion, and chemoresistance. The calcium signaling pathway, the fourth enriched pathway in JIMT-1 cells, can precisely regulate many master regulators in cancer *via* the opening of plasmalemmal calcium channels in response to various stimuli leading to localized high levels of Ca2+, which subsequently influences gene expression (Wu et al., 2021). Ion channels are the key regulators of cellular functions, conducting ions selectively through a pore-forming structure located in the plasma membrane, protein-protein interactions being one of their main regulatory mechanisms.

In line with previously reported data, we observed DAC-induced hypermethylation, accompanied by inverse gene expression in both cell lines. In parallel, DAC-induced down-regulation of both *TET1* and *TET2* genes was identified in the JIMT-1 cell line (log2FC = -1.1 for both genes). TET proteins, differentially expressed in many cancers, are members of the dioxygenase protein family that catalyze the oxidation of 5-methylcytosine to 5-hydroxymethylcytosine. Apart from this canonical function, several alternative modes of TET1-dependent loci- and cell-specific gene regulation have been identified (Filipczak et al., 2019). *TET1* has been reported both as an oncogene and a TSG in human cancers, where its epigenetic inactivation has been demonstrated (Li et al., 2016; Ma et al., 2021). In BC, TET1 was shown to play controversial roles mediated by two isoforms having distinct expression patterns and different functions in tissue development and disease (Alzahayqa et al., 2022). Loss of *TET1* expression due to DAC treatment has also been found in chronic lymphocytic leukemia cells (Kopparapu et al., 2017) and can be at least partially responsible for DAC-induced hypermethylation. Down-regulation of *ESR1* in ER-positive T-47D cells was consistent with the finding of Shenker et al., who showed that DAC treatment (1 µM for 7 days) increased *ESR1* expression in ER-negative cells but significantly reduced intragenic methylation and expression of *ESR1* in ER-positive cells, including T-47D cell line (Shenker et al., 2015). These results revealed that molecular particularities of cells could substantially affect the susceptibility to DAC treatment.

Interestingly, two common pathways were oppositely regulated in JIMT-1 and T-47D cells. First, the Rap1 signaling pathway controls diverse processes, such as cell adhesion, cell-cell junction formation, and cell polarity. The focal adhesion pathway regulates cell motility, cell proliferation, cell differentiation, regulation of gene expression, and cell survival. Both pathways are closely related to invasion and metastasis (Zhang et al., 2017; Sacks et al., 2018).

Given the unspecific action and broad range of DNMT inhibitors, the chance that they could reactivate oncogenes or silence TSGs has to be considered despite their positive effects. In agreement with previously published results, we found a relatively small number of up-regulated oncogenes or down-regulated TSG. Turchinovich et al. identified 18 out of 48 most common oncogenes and 9 out of 46 TSGs deregulated in DAC-exposed cells, while expression changes of only three of the oncogenes and four TSGs were unambiguously attributed to CpG hypomethylation (Turchinovich et al., 2019). In the context of large-scale epigenomic reprogramming in healthy tissues, the impact of low DAC doses (up to 0.35 mg/kg administered three times a week for 7 weeks) was deeply characterized in mice organs (Colwell et al., 2021). The authors concluded that DNA methylation and gene expression were disrupted *in vivo* in a non-uniform manner and that no dose level or regimen is sufficient to cause systemic hypomethylation.

The main limitation of the first- and second-generation epi-drugs, including DAC, is their poor bioavailability, low stability, short half-life, and broad-scale reprogramming. In addition, their efficacy has been strongly influenced by inappropriate administration and cell toxicity (Nervi et al., 2015). Moreover, low proliferation activity could be the major obstacle to their use in the therapy of solid tumors (Graça et al., 2016). Therefore, novel strategies are required to increase the efficacy and safety of this group of epi-drugs. The common side effects of nucleoside analogs are mutagenic risk and genomic instability that could be avoided using non-nucleoside analogs (such as hydralazine, procainamide, RG108, and MG98) (Cheng et al., 2019). However, the essential precondition for increasing the effectiveness and safety of epi-drugs, either alone or in combination, is a deeper understanding of their molecular mode of action and complex interactions.

## Data Availability

The data presented in the study are deposited in the ArrayExpress repository, accession numbers E-MTAB-12224 (RNA-seq) and E-MTAB-12225 (DNA methylation).
