# Supplementary figures and images for: Decitabine-induced DNA methylation-mediated transcriptomic reprogramming in human breast cancer cell lines; the impact of DCK overexpression
Source: Front Pharmacol. 2022 Oct 5;13:991751. doi: 10.3389/fphar.2022.991751 (PMC9585938; doi:10.3389/fphar.2022.991751)

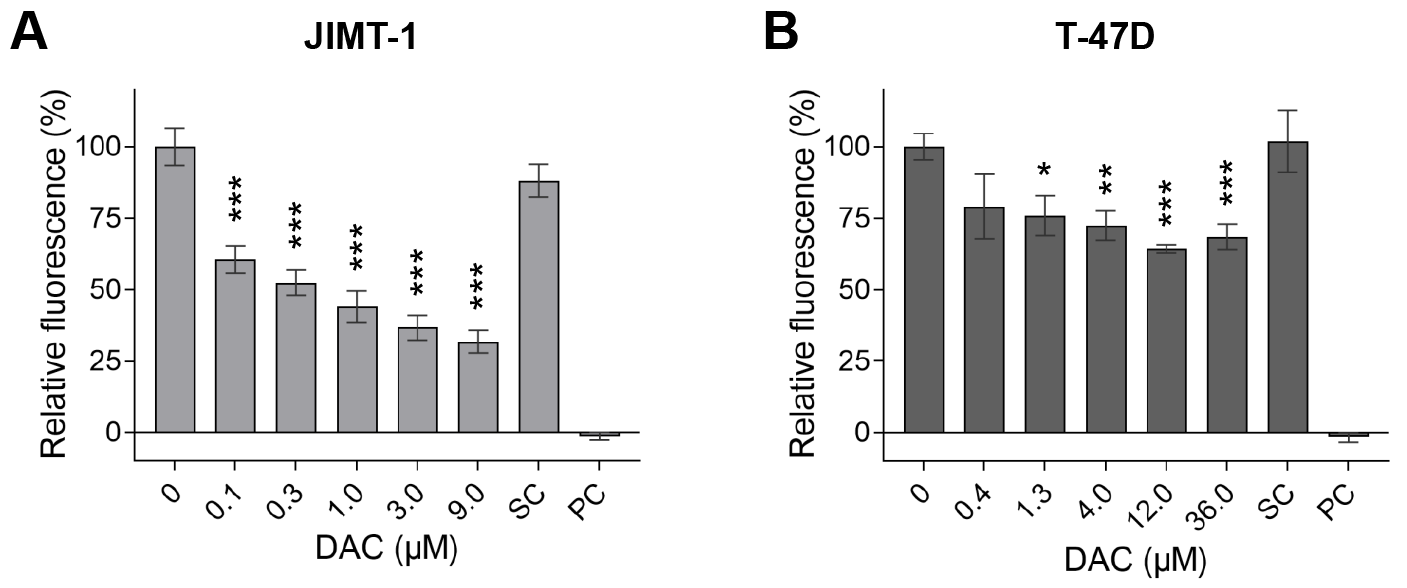

Supplement: Supplementary file 2 [file Image2.tif]

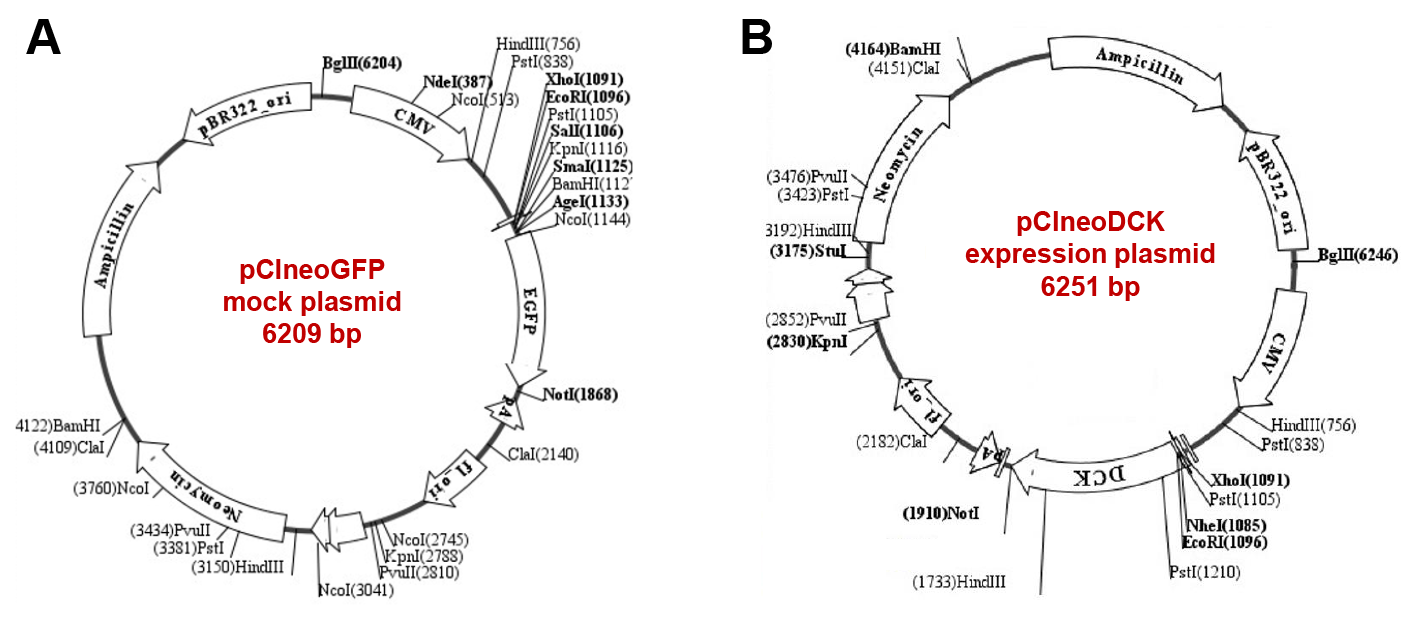

Supplement: Supplementary file 3 [file Image1.tif]
