# Supplementary material for: Decitabine-induced DNA methylation-mediated transcriptomic reprogramming in human breast cancer cell lines; the impact of DCK overexpression
Source: Front Pharmacol. 2022 Oct 5;13:991751. doi: 10.3389/fphar.2022.991751 (PMC9585938; doi:10.3389/fphar.2022.991751)
Supplement: Supplementary file 5 [file DataSheet1.docx]

Supplementary Material


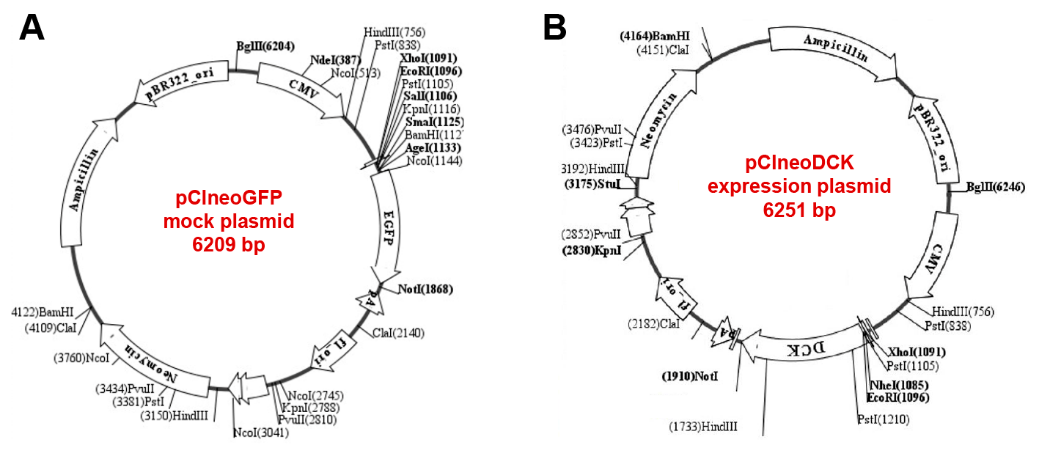


**Supplementary Figure 1.** Sequence map for (A) pCIneoGFP and (B) pCIneoDCK vectors.


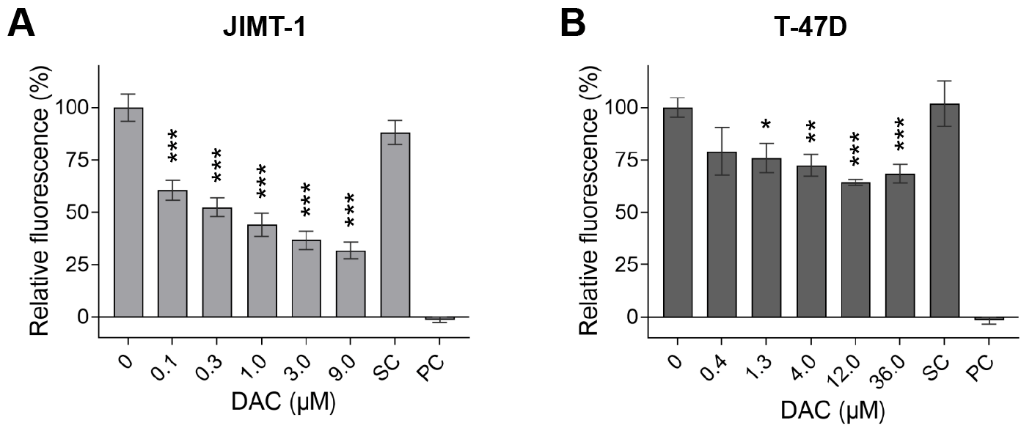


**Supplementary Figure 2.** Cell viability investigated by the Alamar blue assay on **(**A) JIMT-1 and (B) T-47D cells expressed as mean ± SEM. Differences to untreated cells; * p < 0.05, ** p < 0.01, *** p < 0.001. Abbreviations: SC – solvent control, PC – positive control.
